# Hybrid Chiral MoS_2_ Layers for Spin‐Polarized Charge Transport and Spin‐Dependent Electrocatalytic Applications

**DOI:** 10.1002/advs.202201063

**Published:** 2022-04-28

**Authors:** Zhiyun Bian, Kenichi Kato, Tomoki Ogoshi, Zhou Cui, Baisheng Sa, Yusuke Tsutsui, Shu Seki, Masayuki Suda

**Affiliations:** ^1^ Department of Molecular Engineering Graduate School of Engineering Kyoto University Nishikyo‐ku Kyoto 615‐8510 Japan; ^2^ Department of Synthetic Chemistry and Biological Chemistry Graduate School of Engineering Kyoto University Nishikyo‐ku Kyoto 615‐8510 Japan; ^3^ Key Laboratory of Ecomaterials Advanced Technology College of Materials Science and Engineering Fuzhou University Fuzhou 350108 P. R. China; ^4^ JST‐PRESTO Honcho 4‐1‐8 Kawaguchi Saitama 332‐0012 Japan

**Keywords:** chiral MoS_2_, CISS effect, spin‐dependent electrochemistry, spin‐polarized current, spintronics

## Abstract

The chiral‐induced spin selectivity effect enables the application of chiral organic materials for spintronics and spin‐dependent electrochemical applications. It is demonstrated on various chiral monolayers, in which their conversion efficiency is limited. On the other hand, relatively high spin polarization (SP) is observed on bulk chiral materials; however, their poor electronic conductivities limit their application. Here, the design of chiral MoS_2_ with a high SP and high conductivity is reported. Chirality is introduced to the MoS_2_ layers through the intercalation of methylbenzylamine molecules. This design approach activates multiple tunneling channels in the chiral layers, which results in an SP as high as 75%. Furthermore, the spin selectivity suppresses the production of H_2_O_2_ by‐product and promotes the formation of ground state O_2_ molecules during the oxygen evolution reaction. These potentially improve the catalytic activity of chiral MoS_2_. The synergistic effect is demonstrated as an interplay of the high SP and the high catalytic activity of the MoS_2_ layer on the performance of the chiral MoS_2_ for spin‐dependent electrocatalysis. This novel approach employed here paves way for the development of other novel chiral systems for spintronics and spin‐dependent electrochemical applications.

## Graphical abstract

Chiral‐induced spin selectivity in the chiral MoS_2_ is reported. The spin selectivity suppresses the production of the H_2_O_2_ by‐product and promotes the formation of the ground state O_2_ molecules during the oxygen evolution reaction. The synergistic effect of the high spin‐polarization and the high catalytic activity of the MoS_2_ layer for spin‐dependent electrocatalysis is successfully demonstrated.

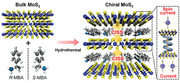

## 1 Introduction

In spintronics, charge and spin: the degree of freedom of electrons are used to carry and store information. Spin‐polarized currents are commonly generated through the application of an external magnetic field to inorganic ferromagnetic materials and materials that exhibit strong spin–orbit interactions (SOIs).^[^ ^1^, ^2^, ^3^, ^4^, ^5^ ^]^ Due to the weak SOI of light elements, organic materials are deemed unsuitable for spin current generation.^[^ ^6^, ^7^, ^8^, ^9^, ^10^ ^]^ The chiral‐induced spin selectivity (CISS) effect in chiral molecules was, however, game‐changing discovery opening up the possibility of utilizing organic materials for spintronic applications within these two decades. Through the CISS effect, chiral organic molecules can act as spin filters that transmit electrons into preferential spin orientations.^[^ ^11^, ^12^ ^]^ Apart from spintronic applications, the CISS effect has also proven its utility in enantioseparation^[^ ^13^, ^14^ ^]^ and spin‐dependent electrochemical procedures.^[15–^ ^19^ ^]^ Most of the reports on the CISS effect were limited to measure tunneling currents through monolayers of chiral molecules, such as DNA,^[^ ^20^, ^21^ ^]^ helicenes,^[^ ^22^ ^]^ oligopeptides,^[^ ^23^ ^]^ and chiral molecule‐capped quantum dots.^[^ ^24^ ^]^ Although the microscopic mechanism of the CISS effect is still under discussion, spin selectivity has been verified through various physical property measurement techniques, such as X‐ray circular dichroism (CD) measurements,^[^ ^25^ ^]^ spin‐polarized conductive atomic force microscopy (c‐AFM),^[^ ^26^, ^27^ ^]^ magnetoresistance measurements,^[^ ^28^ ^]^ and single‐molecule scanning tunnel microscopy.^[^ ^23^ ^]^

The maximum spin polarization (SP) through the chiral monolayer at room temperature was ≈60%,^[^ ^20^ ^]^ which was higher than those of general transition metal ferromagnets and their alloys (up to 45%).^[^^]^ 1D supramolecular nanofibers, 2D chiral perovskites and 3D chiral metal–organic frameworks (MOFs) have been reported as ideal spin filters with SP values as high as 85,^[^ ^27^ ^]^ 94,^[^ ^30^ ^]^ and ≈100%,^[^ ^31^ ^]^ respectively. However, these materials are often not used for spintronic or spin‐dependent electrochemical applications due to their low electronic conductivities. The high SP was also reported with several metallic chiral crystal such as CrNb_3_S_6_,^[^ ^32^ ^]^ NbSi_2_, and TaSi_2_;^[^ ^33^ ^]^ however, the number of metallic chiral crystal is limited. On the other hand, inorganic materials‐based chiral catalysts have been reported for several systems, such as chiral molecule functionalized TiO_2_,^[^ ^16^ ^]^ Fe_3_O_4_,^[^ ^17^ ^]^ chiral CuO^[^ ^18^ ^]^ and CoO.^[^ ^19^ ^]^ These chiral systems show good oxygen evolution reaction (OER) performances while its electrical conductivity and SP value are not so high.

**Scheme 1:**
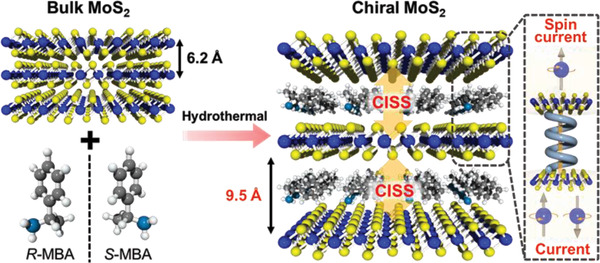
Illustration of the chiral MoS_2_ with multiple CISS effect.

In this study, a novel system with high SP and high electronic conductivity was designed by introducing molecular chirality into layered transition metal dichalcogenides (TMDs). Recently, 2D TMDs with the general formula MX_2_ (M = transition metal, X = chalcogen) have received considerable attention due to their versatile physicochemical properties and unique layered structures with sufficient van der Waals interactions.^[^ ^34^, ^35^, ^36^ ^]^ In particular, molybdenum disulfide (MoS_2_) has been studied extensively as an electronic material and an electrocatalyst for both hydrogen evolution reaction and OER in the water‐splitting process.^[^ ^37^ ^]^ Hexagonal MoS_2_ (2H‐MoS_2_) is a thermodynamically stable semiconductor with an indirect bandgap of 1.2 eV. It can transfer to the octahedral 1T phase (1T’‐MoS_2_) through the efficient intercalation of various guest ions or molecules (Lewis bases) into van der Waals gaps. This first‐order phase transition confers metallic conductivity to MoS_2_ and enhances its electrochemical activity.^[^ ^38^ ^]^ In this context, multilayer 1T'‐MoS_2_ intercalated with chiral molecules is a promising material for spin‐polarized current generators in spintronics and for further electrochemical applications. The electrons conducted through this multilayered structure undergo multiple tunneling processes through the chiral molecules, that is, multiple CISS effects are activated in the material (**Scheme** **1**). The spin relaxation in each multilayer is almost negligible because the monolayer thickness of MoS_2_ (up to a few Å) is far shorter than the spin diffusion length (tens to hundreds of nanometers). In this study, the impact of the activation of multiple CISS mechanisms on the SP of the multilayered material is investigated. Furthermore, the synergistic effect of the possibly high SP and the good catalytic activity of MoS_2_ could potentially facilitate spin‐dependent electrochemical reactions in the multilayered material. As such, the OER performance of the 1T'‐MoS_2_ intercalated with chiral molecules was evaluated as an electrochemical electrode.

## 2 Material Characterization of the Chiral MoS_2_

**Figure 1:**
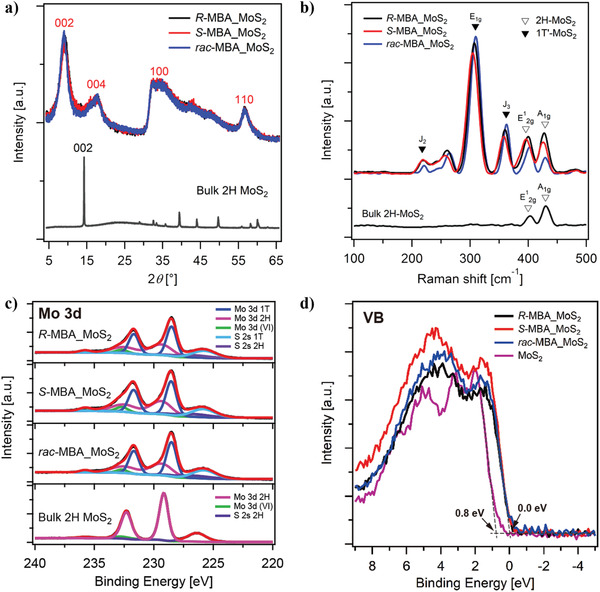
Structural characterization and surface chemistry analysis of the MoS_2_‐based samples. a) XRD patterns, b) Raman spectra, c) Mo 3d and S 2s core‐level regions of the XPS profiles, and d) valence band spectra of the *R/S/rac*‐MBA_MoS_2_ and bulk 2H‐MoS_2_.

The intercalated complexes of chiral MoS_2_ embedded with methylbenzylamine (MBA) molecules were synthesized through a one‐step hydrothermal method. The chiral complexes were produced using (R)‐(+)‐*α*‐methylbenzylamine, (S)‐(‐)‐*α*‐methylbenzylamine, and racemic methylbenzylamine, and labeled as *R*‐MBA_MoS_2_, *S*‐MBA_MoS_2_, and *rac*‐MBA_MoS_2_, respectively. On the other hand, these three chiral complexes are collectively referred to as *R/S/rac*‐MBA_MoS_2_. The X‐ray diffraction (XRD) patterns in **Figure** **1**a reveal that the (002) plane of the *R/S/rac*‐MBA_MoS_2_ and bulk MoS_2_ were observed at 2*θ *= 9.1° and 14.4°, respectively. Correspondingly, the calculated values of the interlayer spacing were 9.5 and 6.2 Å. As such, the intercalation of the chiral MBA molecules increased the interlayer spacing between the *R/S/rac*‐MBA_MoS_2_ layers along the *c*‐axis. The peak width for the (002) plane was slightly sample dependent despite the careful control of the reaction conditions. As described later, the peak width for the (002) plane was highly sensitive to the pH value. This small sample dependence might be originated from the unavoidable differences in the pH value during the reactions. On the other hand, the broad peaks observed at 2*θ *= 32.5° and 56.5° can be attributed to the (100) and (110) planes, respectively. The lattice constant (*a*) of the *R/S/rac*‐MBA_MoS_2_ was recorded at 3.27 Å, which corresponds well to that of the metallic 1T‐MoS_2_ phase (*a* = 3.27 Å).^[^ ^39^ ^]^ The smaller value of *a* of the bulk MoS_2_ was observed for 3.16 Å, which agrees well with that of the semiconducting 2H‐MoS_2_ phase.

The phase compositions of the chiral MoS_2_ samples after the intercalation of the MBA molecules were studied more comprehensively through Raman measurements and X‐ray photoelectron spectroscopy (XPS). The peaks observed at the Raman spectra of the bulk MoS_2_ and the *R/S/rac*‐MBA_MoS_2_ samples at 383 (E^1^_2g_ mode) and 408 cm^−1^ (A^1^_g_ mode) can be ascribed to the in‐plane vibration of the S and Mo atoms, and the out‐of‐plane vibration of the S atoms in the 2H phase, respectively (Figure 1b). The peaks observed at ≈201 (J_2_ mode) and 338 cm^−1^ (J_3_ modes), which correspond to the longitudinal vibration modes of the Mo and S atoms, respectively, confirm the presence of the 1T' phase. Meanwhile, the peak at 283 cm^−1^ is a characteristic of the E_1g_ mode of the 1T' phase. This peak corresponds to the octahedral coordination of the Mo atoms.^[^ ^40^ ^]^ The observed slight shift in the Raman peaks of the *R/S/rac*‐MBA_MoS_2_ is possibly due to the difference in the thicknesses of the layers.^[^ ^41^ ^]^

Figure 1c and Figure S1a in the Supporting Information shows the Mo 3d and S 2p core‐level regions of the XPS profiles of the samples. For the bulk 2H‐MoS_2_ sample, the peaks observed at 232.3 and 229.2 eV (pink line) can be ascribed to Mo^4+^ 3d_3/2_ and Mo^4+^ 3d_5/2_, respectively. Meanwhile, the peak at 226.4 eV (purple line) corresponds to the S 2s species in MoS_2_. The XPS spectra of the *R/S/rac*‐MBA_MoS_2_ can be deconvoluted into five components. Two main components for the 1T″‐MoS_2_ phase were observed with the peaks at 231.7, 228.5 (blue line), and 225.7 eV (right‐blue line) assigned to Mo^4+^ 3d_3/2_, Mo^4+^ 3d_5/2_, and S 2s, respectively, while other two minor components from the 2H‐MoS_2_ phase (pink and purple line) were also observed at almost same peak position with the bulk 2H‐MoS_2_ (details are summarized in Table S1, Supporting Information). Another small component (green line) can be assigned to Mo^6+^ impurity (originated from the precursor material). For the S 2p core level region, other peaks corresponding to the 1T″‐MoS_2_ phase were also observed at 161.3 and 162.6 eV, which can be attributed to S 2p_3/2_ and S 2p_1/2_, respectively. The peaks in the 2H‐MoS_2_ phase were recorded, in contrast, at 162.5 and 163.7 eV assigned to the S 2p_3/2_ and S 2p_1/2_ species (Figure S1a, Supporting Information). Tables S1 and S2 in the Supporting Information summarize the peak positions recorded from the Mo 3d and S 2p core‐level regions with the quantitative analysis on the peaks, respectively. The quantitative analyses confirm the coexistence of 1T' and 2H phases in the *R/S/rac*‐MBA_MoS_2_. This is possibly because the intercalation of the chiral MBA molecules into MoS_2_ was accompanied by rapid electron transfer, which in turn facilitated the transition from the 2H to 1T' phase.^[^ ^42^ ^]^ The ratio of 1T' and 2H phases were determined as ≈5:3.

Figure 1d shows the X‐ray valence band spectra of the bulk MoS_2_ and the *R/S/rac*‐MBA_MoS_2_. The positions of the valence band edge were evaluated through the linear extrapolation of the onset recorded in each spectrum. As a consequence of the intercalation of the MBA molecules, the valence band maxima shifted from 0.8 to 0 eV. This further confirms the transition from the semiconducting 2H phase to the metallic 1T' phase. Along with the phase transition from 2H to 1T’ phase, the electrical conductivity of *R/S/rac*‐MBA_MoS_2_ increased by two orders of magnitude as compared with that of 2H‐MoS_2_ (Figure S2, Supporting Information). These results indicate the successful synthesis of the chiral, multilayered 2H/1T' MoS_2_. Apart from the optical activity, the basic material properties of the *R/S/rac*‐MBA_MoS_2_ samples were undifferentiated. This observation suggests that the synthesis method employed in this study yielded highly reproducible results.

The interlayer distances and total energies of 12 optimized structures of MoS_2_/MBA/MoS_2_ (Figure S3, Supporting Information) were calculated through density functional theory (DFT‐D2) calculations using the Quantum Atomistix ToolKit package. To determine the horizontal or vertical intercalated direction of the MBA molecules among the MoS_2_ layers, three structures each for *R*‐MBA, *S*‐MBA, and *rac*‐MBA, and two types each for the 2H‐ and 1T'‐MoS_2_ monolayers were analyzed. Table S3 in the Supporting Information summarizes the results obtained from the DFT‐D2 calculations. Figure S4 in the Supporting Information shows the fat band structures plotted for the 12 MoS_2_/MBA/MoS_2_ optimized structures. The red and orange parts represent the contribution of the MBA molecules and the MoS_2_ layer, respectively. The MBA molecules prefer to be intercalated horizontally in the MoS_2_ monolayers because the total energies of all the horizontal‐type structures were lower than those of the vertical‐type structures. The obtained interlayer distances (9.49–9.86 Å) from the DFT calculations were in good agreement with the experimental value (≈9.5 Å) obtained through the XRD measurements and transmission electron microscopy (TEM).

## 3 Morphology and Optical Activities of the Chiral MoS_2_

**Figure 2:**
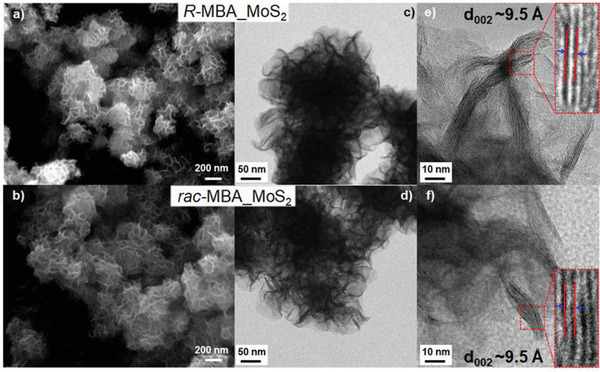
Morphologies of the MoS_2_‐based products synthesized using neutral precursor solutions. a,b) SEM, c,d) TEM, and e,f) HRTEM images of the *R‐*MBA_MoS_2_ and *rac‐*MBA_MoS_2_ samples.

The pH of the precursor solution can considerably affect the morphology and optical activity of the chiral MoS_2_ samples. **Figure** **2** shows the scanning electron microscopy (SEM) and high‐resolution transmission electron microscopy (HRTEM) images of the *R*‐MBA_MoS_2_ and *rac‐*MBA_MoS_2_ synthesized using neutral precursor solutions. The neutral pH was achieved by adding 10 µL HCl. Both samples exhibited flower‐like morphologies with an average diameter of 200 nm; the flower‐like nanocrystals were composed of aggregated thin nanosheets (Figure 2a,b). The TEM (Figure 2c,d) and HRTEM (Figure 2e,f) images further reveal that the structures were formed by multiple bundles of local ordered sheets. The average interlayer spacing was 9.5 Å calculated from a population of 100 distances (Figure S5, Supporting Information). This value corresponds to that obtained from the XRD analysis, and as well from the DFT. Figures S6 and S7 in the Supporting Information show XRD patterns and SEM/TEM images of the *R*‐MBA_MoS_2_ prepared using basic (prepared using 0 µL of HCl) and acidic (prepared using 50 µL of HCl) precursor solutions. At basic conditions, the samples obtained were highly crystalline. Moreover, the nanoparticles formed (average diameter = 500 nm) were larger than those produced using a neutral solution. In contrast, at acidic conditions, the flower‐like morphology was not observed for the synthesized *R*‐MBA_MoS_2_ nanocrystals. Furthermore, the samples prepared under such conditions did not exhibit any optical activity in the CD measurement. The chiral amines from the MBA molecules with p*K*_a_ = 11 possibly increased pH of the solution and in turn suppressed reduction reaction, MoS_4_^2–^ + 2RNH_3_^+^ → MoS_2_ + S + H_2_S + RNH_2_, which yielded bulk MoS_2_ phase. On the other hand, HCl facilitated the formation of the aggregated thin MoS_2_ nanosheets through the reaction MoS_4_^2–^ + 2H^+^ → MoS_2_ + H_2_S + S. In both reactions, MoS_4_^2–^ ions originated from the thermal degradation of the (NH_4_)_2_MoS_4_ precursor. As such, the addition of HCl to adjust the pH of the precursor solution was essential for the formation of the MoS_2_ nanostructures, which show optical activity.^[^ ^43^ ^]^ Therefore, we focused onto the products synthesized using near‐neutral precursors.

**Figure 3:**
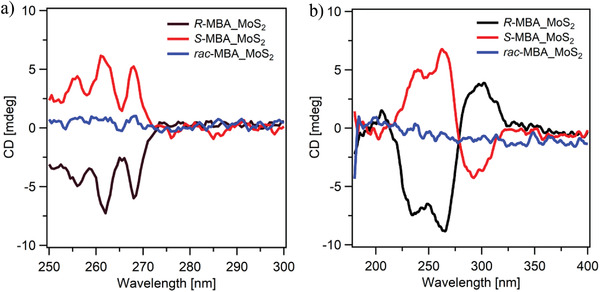
CD spectra of the a) *R/S/rac*‐MBA solutions and b) *R/S/rac*‐MBA_MoS_2_ thin films.

The chiroptical properties of the *R/S/rac*‐MBA solutions and *R/S/rac*‐MBA_MoS_2_ samples were studied through transmission CD measurements. Symmetric signals at 256, 262, and 268 nm were observed at the CD spectra of the *R*‐MBA and *S*‐MBA solutions. In contrast, no CD signal was recorded on the spectrum of the *rac*‐MBA solution (**Figure** **3**a). The CD spectra of the *R/S/rac*‐MBA_MoS_2_ samples were recorded using thin films deposited on quartz substrates. The nanocrystals were dispersed in dimethylformamide (DMF), and the resulting solutions were drop casted on the substrates. The CD spectra of the *R*‐MBA_MoS_2_ and *S*‐MBA_MoS_2_ thin films exhibited symmetric CD signals at similar wavelengths (206, 240, 263, and 298 nm). On the other hand, no CD signal was observed at the spectrum of the *rac*‐MBA_MoS_2_ film (Figure 3b). Furthermore, the positions of the CD signals recorded for the *R*‐MBA_MoS_2_ and *S*‐MBA_MoS_2_ thin films were shifted significantly from those observed for the *R*‐MBA and *S*‐MBA solutions. The cotton effect was observed for the chiral MoS_2_ thin films at 213 and 279 nm, wherein the sign of the CD signal inverted across the zero line. This can be attributed to the splitting of the energy levels after the intercalation of the chiral MBA molecules.^[^ ^44^ ^]^ Therefore, the observed CD signal in the *R* and S ‐MBA_MoS_2_ thin films are not caused by only chiral MBA molecules but caused by the hybrid chiral systems.

## 4 Spin‐Dependent Charge Transport Properties by Spin‐Polarized c‐AFM Measurements

**Figure 4:**
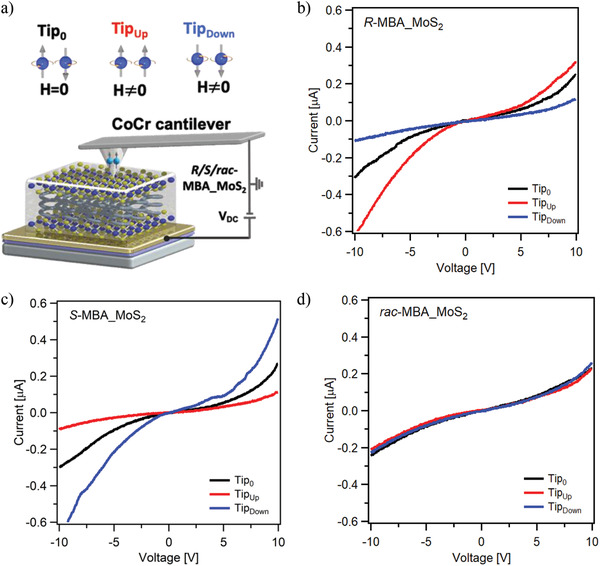
Spin‐polarized c‐AFM measurements. a) Schematic of the experimental setup. *I–V* curves from −10 to +10 V of the chiral, b) *R‐*MBA_MoS_2_, c) *S*‐MBA_MoS_2_, and d) *rac*‐MBA_MoS_2_ thin films. The CoCr tip used was either nonmagnetized (black) or magnetized along the upward (red) or downward (blue) orientations. The average *I–V* curve recorded over 40 scans at different points is shown.

The spin‐dependent charge transport properties of the *R/S/rac*‐MBA_MoS_2_ films were investigated through spin‐polarized c‐AFM using ferromagnetic CoCr tips. The *R/S/rac*‐MBA_MoS_2_ thin films were prepared by drop casting corresponding DMF solutions onto quartz substrates initially sputtered with Ti (6 nm) and Au (50 nm) layers. The current–voltage (*I−V*) plots were recorded at different positions with at least 40 traces in contact mode either with the nonmagnetized (*H* = 0) or magnetized (*H* ≠ 0) CoCr tips along the upward (Tip_Up_) and downward (Tip_Down_) orientations. Figure S9 in the Supporting Information shows the raw data obtained. An electric bias voltage was applied between the CoCr tip and the gold substrate (**Figure** **4**a). Figure 4b–d shows the average *I–V* curves of the *R/S/rac*‐MBA_MoS_2_ films. For the *R*‐MBA_MoS_2_ thin film, the values of the current from −10 to +10 V recorded under the Tip_Up_ condition were higher significantly than those measured using the Tip_Down_ setting of the tips (Figure 4b). This reveals that the chirality of the *R*‐MBA_MoS_2_ thin film can preferentially select up‐spin charge carriers and separate the down‐spin ones. The contrary was observed for the *S*‐MBA_MoS_2_ films (Figure 4c); higher currents were recorded when the tip was magnetized downward. Lastly, no considerable changes on the current readings of the *rac*‐MBA_MoS_2_ film were observed at different tip magnetization settings (Figure 4d). The SP was evaluated for the polarized currents using the formula

(1) $$\mathrm{SP}\left(\%\right)=\frac{I_{\mathrm{Up}}-I_{\mathrm{Down}}}{I_{\mathrm{Up}}+I_{\mathrm{Down}}}\times 100\%$$

where *I*_Up_ and *I*_Down_ are the currents measured at −10 V when the CoCr tip was magnetized along the upward and downward orientations, respectively. From the spin‐polarized c‐AFM results, the calculated SP for the *R‐*MBA_MoS_2_ and *S*‐MBA_MoS_2_ films were +71 and −75%, respectively. Figure S10 in the Supporting Information shows that the SP of *R‐*MBA_MoS_2_ and *S*‐MBA_MoS_2_ increased gradually with increasing bias voltage and finally reached +71% and −75% at −10 V.

Most of the previous reports on the CISS effect were limited to the measurement of the tunneling current through various chiral monolayers and length‐dependent chiral systems. The maximum SP achieved for molecular monolayer was ≈60%.^[^ ^20^ ^]^ Therefore, considerable improvements in the spin polarization that rely solely on the design of chiral molecules are often difficult to achieve. Here, the current repeatedly flowing through the multiple layers of MoS_2_ intercalated with chiral molecules activated multiple CISS effects in the material. As such, a relatively high SP close to 75% was obtained. The improved SP may be attributed to the multiple spin‐polarization processes (i.e., multiple CISS processes) through the chiral organic molecules that were formed in the MoS_2_ multilayers (Scheme 1). In addition, in this system, the short chiral MBA molecules were used to control the interlayer distance and ensure the occurrence of the tunneling process. These preserve the high conductivity of the MoS_2_ layers and facilitate the achievement of a high SP through the multiple CISS mechanism. Indeed, the SP increased with increasing the transport distance (film thickness) as shown in Figure S11 in the Supporting Information. These results clearly indicate that the electrons conducted through this multilayered structure undergo multiple tunneling processes through the chiral molecules and the SP was enhanced by repeating the tunneling processes.

## 5 Electrochemical Measurements

### 5.1 OER Study

**Figure 5:**
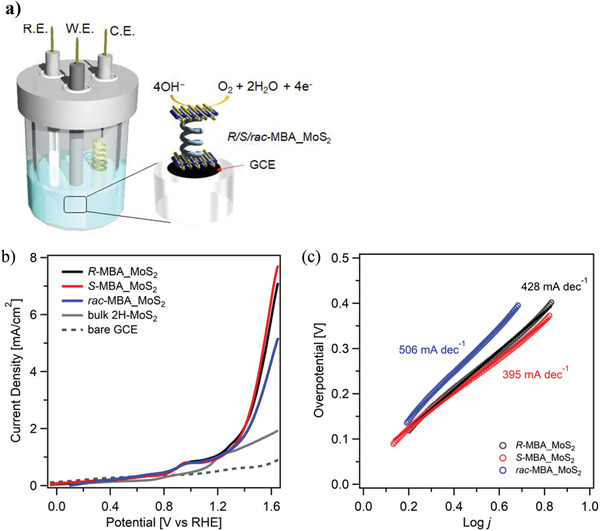
Performance of the *R/S/rac‐*MBA_MoS_2_ and the bulk 2H‐MoS_2_ samples as electrocatalysts for OER. a) Schematic of the three‐electrode cell setup. b) LSV curves from 0 to 1.6 V in 0.1 m KOH recorded at a scan rate of 10 mV s^–1^ and the c) corresponding Tafel plots of the *R/S/rac‐*MBA_MoS_2_. The solid lines show linear fit to the data. The details of fitting are shown in Figure S13 in the Supporting Information.

**Figure** **5**a shows the standard three‐electrode cell setup used for the electrochemical water‐splitting measurements. The reference and counter electrodes used were Ag/AgCl (saturated NaCl) and Pt wire, respectively. The electrochemical studies were carried out using a 0.1 m KOH (pH = 13) aqueous electrolyte. The working electrode was prepared depositing the *R*/*S*/*rac*‐MBA_MoS_2_ samples onto a glassy carbon (GC) electrode. The activities of the *R‐*MBA_MoS_2_, *S*‐MBA_MoS_2_, and *rac*‐MBA_MoS_2_ electrodes toward OER were studied through linear sweep voltammetry (LSV). The LSV curves in Figure 5b show that the current densities recorded using the *R‐*MBA_MoS_2_ and *S*‐MBA_MoS_2_ anodes were higher than those achieved using the *rac*‐MBA_MoS_2_ anode. For comparison, the LSV curve of the bare GC electrode revealed the current collector used had poor OER catalytic activity. Prior to the onset of OER, low intensity peaks were observed, which can be attributed to the presence of highly active impurities in the electrolyte.^[^ ^45^ ^]^ The kinetics of the OER process were evaluated using the Tafel equation, *η* = *a* + *b* log_10_(*j*), where *a, b, j*, and *η* are the intercept, Tafel slope (mV dec^–1^), current density and overpotential, respectively (Figure S13, Supporting Information). The Tafel slopes decreased in the order: 506 mV dec^–1^ (*rac*‐MBA_MoS_2_) >428 mV dec^–1^ (*R*‐MBA_MoS_2_) >395 mV dec^–1^ (*S*‐MBA_MoS_2_) (Figure 5c). This indicates that the chiral MoS_2_ samples had the faster OER kinetics than the racemic MoS_2_ samples. Although relatively high Tafel slopes compared to other benchmark inorganic chiral electrodes (70–80 mV dec^−1^ for chiral CuO and 101 mV dec^−1^ for chiral CoO) were calculated for the electrodes prepared in this study, the values were lower than those reported for other non‐noble metal‐based OER catalysts such as the MnO*_x_* (653 mV dec^−1^).^[^ ^46^ ^]^ These results demonstrate that the chiral molecules possibly enhances the OER catalytic activity of the MoS_2_ layers. Note that the maximum SP in the current system is ≈75%, however, the SP decreased with decreasing the bias. In the OER experiments, which were performed with the bias below 2 V, the effective SP used for the reaction is ≈50%. In other words, there is still the possibility for enhancement of the OER activity.

### 5.2 H_2_O_2_ Detection

**Figure 6:**
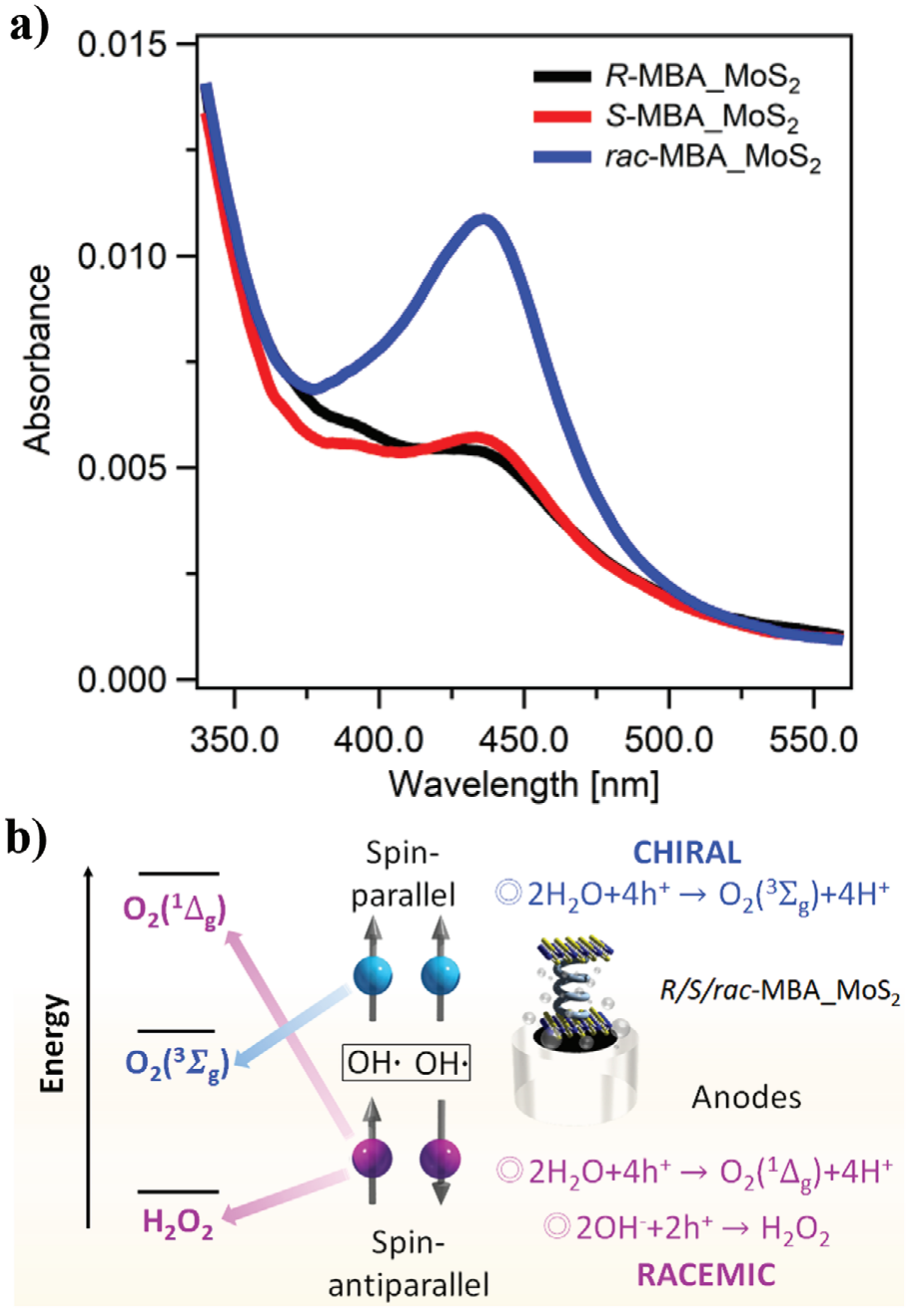
Detection of the produced H_2_O_2_ during OER. a) UV−vis absorption spectra after the titration with *o*‐tolidine of the electrolytes after the electrochemical measurements using chiral and racemic MoS_2_ electrodes. b) Energy diagram of the different mechanistic pathways for spin‐parallel (blue) and spin‐antiparallel (purple) of •OH radicals and the possible reaction products on the chiral and racemic MoS_2_ surfaces.

To verify the role of spin selectivity on the OER catalytic activity of the samples, the formation of H_2_O_2_ by‐product was determined during the water‐splitting process on the chiral and racemic MoS_2_ electrodes. Bulk electrolysis (BE) in coulometry mode with a charge of 0.08 C at a constant voltage of 1.4 V versus Ag/AgCl was performed using a 0.1 m Na_2_SO_4_ solution (pH = 6.56) (Figure S14, Supporting Information). After the BE, the dissolved H_2_O_2_ in the electrolyte was detected through a spectrophotometric titration experiment using *o*‐tolidine as the redox indicator. The reaction of the produced H_2_O_2_ with *o*‐tolidine develops an absorption peak at 437 nm. The absorption maximum became more apparent on the UV–vis spectra of the titrated electrolyte solution obtained after BE using the *rac*‐MBA_MoS_2_ anode (**Figure** **6**a). This indicates the generation of H_2_O_2_ during the OER using the racemic MoS_2_ electrode. The UV–vis results also imply that the chirality of the *R‐*MBA_MoS_2_ and *S‐*MBA_MoS_2_ samples potentially suppressed the formation of the H_2_O_2_ by‐product.

The OH• radicals generated by the chiral MoS_2_ electrodes are aligned in a parallel orientation, which interacts with the triplet potential surface to form the ground state O_2_ molecules (^3^Σ_g_) (Figure 6b). Notably, the production of H_2_O_2_ is spin‐forbidden reaction on the surface of the triplet potential. Therefore, an anticorrelated relationship can be drawn between H_2_O_2_ and triplet O_2_, that is, H_2_O_2_ cannot be generated with O_2_ (^3^Σ_g_). When the spins of electrons are aligned in an antiparallel orientation, the OH• radicals interact at the singlet potential surface, resulting reasonably in the formation of singlet H_2_O_2_ or O_2_ (^1^Δ_g_). The large overpotential encountered during OER has been associated with the formation of O_2_ (^1^Δ_g_) and the kinetically favorable H_2_O_2_ production without spin confinement.^[^ ^16^, ^17^ ^]^ Therefore, the CISS effect can improve the water‐splitting efficiency of electrodes by inhibiting the formation H_2_O_2_ and facilitating the selective production of O_2_ (^3^Σ_g_). In the current system, the high efficiency and activity of the MoS_2_‐based electrodes toward OER was realized by the synergetic effect of the high SP value due to the activation of multiple CISS mechanisms and the high catalytic activity of the MoS_2_ layer.

## 6 Conclusion

In this study, multilayered MoS_2_ intercalated with chiral MBA molecules was successfully synthesized for the first time through a one‐step hydrothermal method. The spin transport in the chiral MoS_2_ (*R*‐MBA_MoS_2_ and *S*‐MBA_MoS_2_) was heavily impacted by the magnetization orientations of the tips used in the c‐AFM measurements and the handedness of the embedded chiral organic molecules. A SP as high as 75% was measured on the hybrid *S*‐MBA_MoS_2_ thin film, possibly due to the activation of multiple spin‐tunneling channels. Moreover, the MoS_2_‐based electrodes exhibited high activities and efficiencies as OER electrocatalysts due to the synergetic effect of the high SP value and high catalytic activity of the MoS_2_ layer. The results reported in the study demonstrate the efficient spin‐dependent charge transport and water splitting in the hybrid chiral MoS_2_. We believe this study will pave a way for the design and development of next‐generation hybrid chiral materials for spintronic and spin‐dependent electrochemical applications.

## 7 Experimental Section

### Synthesis of R/S/rac‐MBA_MoS_2_

Chiral MoS_2_ layered nanostructures were synthesized through the hydrothermal method. (NH_4_)_2_MoS_4_ precursor (66 mg, 0.254 mmol) was dissolved in deionized water (20 mL). The solution was sonicated for 10 min. Then, (R)‐(+)‐*α*‐methylbenzylamine (*R*‐MBA, 32.3 µL), (S)‐(‐)‐*α*‐methylbenzylamine (*S‐*MBA, 32.3 µL), and racemic methylbenzylamine (*rac*‐MBA, 0.254 mmol) were added in the as‐formed solution to obtain the intercalated chiral nanostructures. HCl (37 vol%, 10 µL) was added to disperse the aggregated nanostructures. The reaction mixture was transferred to 50 mL Teflon‐lined stainless‐steel autoclaves. The hydrothermal reaction was carried out at 200 °C for 12 h in an electric oven. The black products were vacuum‐filtered using a polytetrafluoroethylene membrane filter and washed thoroughly with deionized water and acetone several times. The final product was vacuum‐dried at room temperature.

### Material Characterizations

The XRD patterns of the *R/S/rac‐*MBA_MoS_2_ nanostructures were obtained using a MiniFlex600 + D/teX Ultra (Rigaku) diffractometer at 40 kV and 15 mA with a Cu‐*K*_*α*_ radiation source (*λ* = 1.54056 Å). Raman spectroscopy was performed on an NRS‐4100 (JASCO) spectrometer with a 20.1 mW laser (*λ* = 532 nm) from 100–1000 cm^–1^. XPS measurements for Mo 3d, S 2p, N 1s and valence band region were recorded on a JPS‐9010TRX (JEOL) spectrometer with an Al‐*K*_*α*_ X‐ray at 12 kV and 25 mA. The peaks were resolved using the Shirley background and Voigt functions to identify the corresponding bonds. SEM and field‐emission (FE)‐TEM were performed using LSM‐7001F (JEOL) and JEM‐2100F(G5) (JEOL) microscopes, respectively. FE‐TEM was performed at an accelerating voltage of 200 kV using samples prepared on a carbon‐reinforced collodion film 250 square‐mesh copper grid (COL‐C10, Oken Shoji).

### CD Measurements

The CD spectra of the samples were obtained using a JASCO J‐1500 spectrometer. Throughout the measurements, the system was purged with N_2_ gas. To fabricate the test samples, *R/S/rac‐*MBA solutions (1 × 10^−3^ m) were prepared by dissolving *R/S/rac*‐MBA organic molecules (2.5 µL) in ethanol in a 20 mL volumetric flask. For the *R/S/rac‐*MBA_MoS_2_ thin films, the quartz substrates were washed with acetone and isopropanol for 30 min in a sonicator and then treated using an UV‐ozone cleaner for 2 min. The *R/S/rac‐*MBA_MoS_2_ (5 mg) products were dissolved in DMF (1 mL) to prepare the precursor solutions. The resulting mixtures were sonicated for 1 h to completely disperse the powders. Then, the precursor solution (20 µL) was drop casted immediately on the quartz substrate. The films on the quartz substrates were dried at room temperature. CD measurements from 180 to 400 nm were recorded through single accumulation at a scanning speed of 100 nm min^−1^, data interval of 0.2 nm, bandwidth of 1 nm and single accumulation were used from 180 to 400 nm, and the data were presented as the smoothed CD signals using an internal algorithm in the JASCO software package. The reliable CD data were obtained by setting the high‐tension voltage of the photomultiplier tube detector to no more than 800 V. To determine the optical purity of the thin films, the UV−vis absorption spectra from 180–400 nm were simultaneously recorded by the CD spectrometer.

### Spin‐Polarized c‐AFM Measurements

To prepare the samples for the c‐AFM measurements, solutions (5 mg mL^−1^) composed of the *R/S/rac‐*MBA_MoS_2_ powders in DMF solvents were drop casted onto fresh quartz substrates, which were initially sputtered with Ti (6 nm) and Au (50 nm) layers (Nano PVD‐S10A, Nanotechnology, England). These samples were annealed at 100 °C to evaporate the solvent. The typical film thickness was controlled to ≈5 µm. The *I–V* measurements were obtained using an SPA‐400 AFM system with a NanoNavi IIe controller. The CoCr tips (MESP‐V2, Bruker) were premagnetized in different field orientations, such as magnetic north (Tip_Up_), south (Tip_Down_), and no magnetic orientation (Tip_0_), using a strong permanent magnet. Then, these were used immediately for the *I–V* scans of the three samples in contact mode. Relatively flat domains with typical roughness of ≈200 nm were selected and the measurements were recorded for more than 40 times from −10 to +10 V, and the average *I–V* measurements were reported. Each curve was obtained by placing the tip in a new position to avoid damaging the sample.

### Electrical Conductivity Measurements

The 2H‐MoS_2_ and *R/S/rac*‐MBA_MoS_2_ powders (≈5 mg) were pressed into pellets of 5 mm in diameter using MODEL MP‐1 mini‐press (JASCO). Standard four‐probe method measurements were carried out with four gold wires (15 µm*ϕ*) attached with silver paste. Constant current was applied by a 2400 source meter (Keithley), and the potential difference was measured by a 2182A nanovoltmeter (Keithley). All the measurements were performed under vacuum in a variable temperature cryostat (Montana instruments Cryostation).

### Electrochemical Characterization

Electrochemical experiments were conducted in a three‐electrode cell setup with a 0.1 m aqueous KOH solution (pH = 13) as the electrolyte, Ag/AgCl (saturated NaCl) as the reference electrode, and a Pt wire as the counter electrode. The *R/S/rac*‐MBA_MoS_2_ samples (10 mg) were dispersed in Nafion (10 µL) and isopropyl alcohol (0.49 mL). Then, the mixture (20 µL) was deposited onto a GC disk working electrode (area = 0.16 cm^2^). Then, the GC disk was annealed at 100 °C for 30 min to evaporate the solvent. LSV was performed from −0.5 to 1.6 V (vs Ag/AgCl reference electrode) at a scan rate of 10 mV s^−1^. The *R/S/rac*‐MBA_MoS_2_‐coated GC disk electrode was not stable at over 1.6 V. The current density was calculated using the surface area of the original GC electrode.

### H_2_O_2_ Detection

BE measurements in coulometry mode were performed using the *R/S/rac‐*MBA_MoS_2_ samples in a 0.1 m Na_2_SO_4_ aqueous solution at 1.4 V to generate a charge of 0.08 C. Bubbles were formed on the Pt wire and the *R/S/rac*‐MBA_MoS_2_‐coated GC disk electrode due to the evolution of H_2_ and O_2_ during the electrochemical measurements. The dissolved H_2_O_2_ in the Na_2_SO_4_ electrolyte was detected through colorimetric titration, where *o*‐tolidine was used as a redox indicator. After the reaction in the electrochemical cell, the *o*‐tolidine (Sigma‐Aldrich Co., 0.8 mL) indicator was added into the electrolyte solution (4 mL). These components were left to react for 60 min. From the UV–vis spectroscopy, the H_2_O_2_ absorption peak of the rac‐MBA_MoS_2_ solution appeared as a faint yellow color at ≈437 nm.

### Computational Details

All calculations were performed through the DFT‐D2 method using the Quantum Atomistix ToolKit package.^[^ ^47^ ^]^ The exchange correlations were described by the generalized gradient approximation applying Perdew–Burke–Ernzerhof (GGA‐PBE) functional with the PseudoDojo pseudopotential,^[^ ^48^ ^]^ and the double‐z‐polarized basis set embedded in the linear combination of atomic orbitals basis.^[^ ^49^ ^]^ The density mesh cutoff was 80 Hartree and the DFT‐D2 method was employed to deal with the interlayer van der Waals force. A 3×3×1 sampling *k*‐point mesh was employed for the self‐consistent calculation and optimization of the geometry. Y (0, 0.5, 0), Γ (0, 0, 0), X (0.5, 0, 0), and V (0.5, 0.5, 0) were selected to calculate the projected band structure.

### Statistical Analysis

The background subtraction and the peak deconvolution for the XPS spectra were performed using the SpecSurf, which is the original analysis software of the JPS‐9010TRX (JEOL) spectrometer. The other statistical tests in this study were performed using igor PRO (WaveMetrics).

## Conflict of Interest

The authors declare no conflict of interest.

## Supporting information

Supporting Information

## Data Availability

The data that support the findings of this study are available from the corresponding author upon reasonable request.
