# Supplementary material for: Hybrid Chiral MoS2 Layers for Spin‐Polarized Charge Transport and Spin‐Dependent Electrocatalytic Applications
Source: Adv Sci (Weinh). 2022 Apr 28;9(17):2201063. doi: 10.1002/advs.202201063 (PMC9189682; doi:10.1002/advs.202201063)
Supplement: Supplementary file 1 — Supporting Information [file ADVS-9-2201063-s001.pdf]

ADVANCED SCIENCE
Open Access

Supporting Information

for *Adv. Sci.*, DOI 10.1002/advs.202201063

Hybrid Chiral MoS_2 Layers for Spin-Polarized Charge Transport and Spin-Dependent Electrocatalytic Applications

*Zhiyun Bian, Kenichi Kato, Tomoki Ogoshi, Zhou Cui, Baisheng Sa, Yusuke Tsutsui, Shu Seki and Masayuki Suda**

Supporting Information

Hybrid Chiral MoS_2 Layers for Spin-polarized Charge Transport and Spin-dependent Electrocatalytic Applications

*Zhiyun Bian, Kenichi Kato, Tomoki Ogoshi, Zhou Cui, Baisheng Sa, Yusuke Tsutsui, Shu Seki and Masayuki Suda**

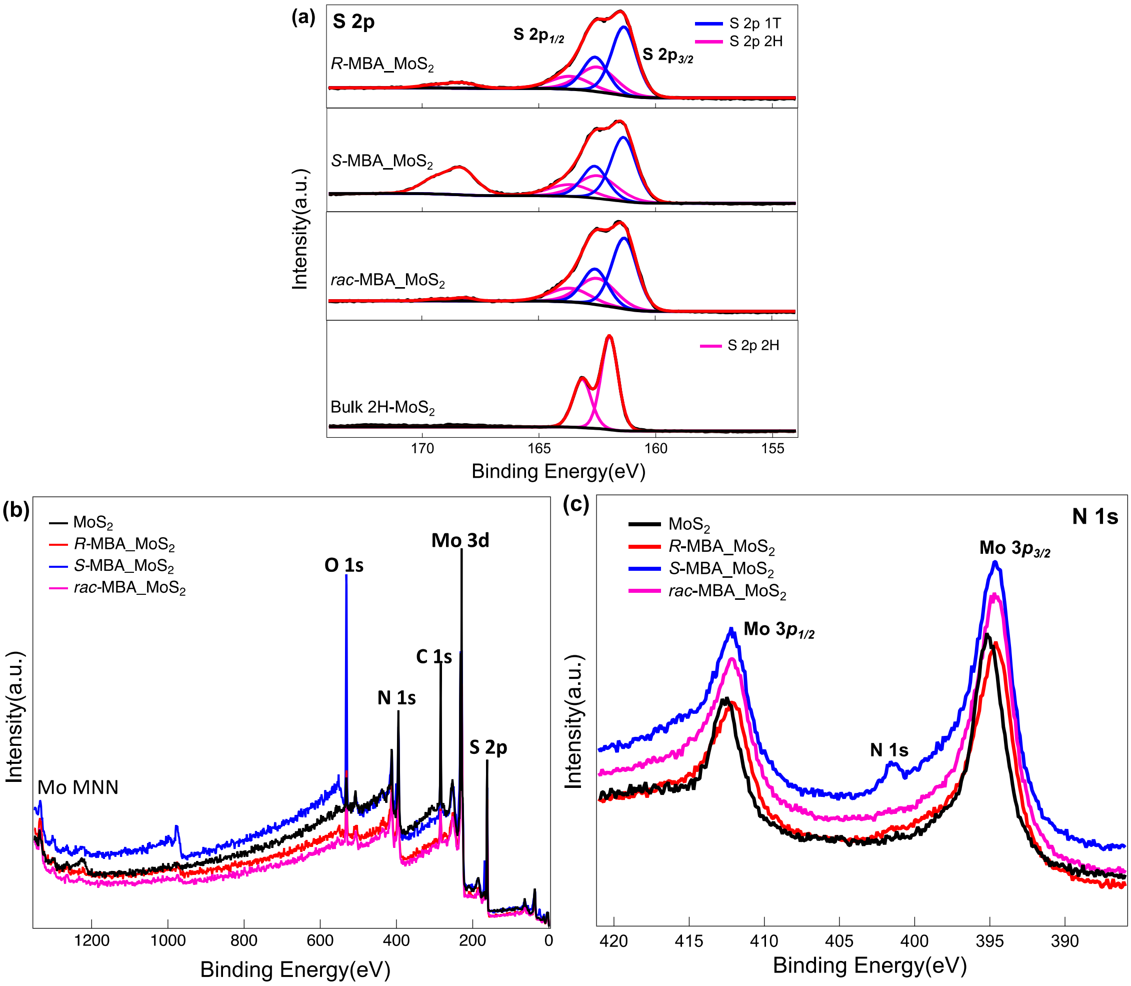

Figure S1. XPS spectra of 2H-MoS_2 and *R/S/rac*-MBA_MoS_2, showing the (a) S 2p, (b) N 1s and (c) survey spectra. The peaks around 169 eV in S 2p spectra can be attributed to S $2p_{1/2}$ (169.4 eV) and S $2p_{3/2}$ (168.2 eV) of the sulfate produced by the oxidation of sulfur during the synthesis. The peak around 401.7 eV in N1s spectra can be attributed to the N1s of the ammonium salt originated from $(NH_4)_2MoS_4$ used as precursor.

Table S1. Peak positions in Mo 3d and S 2p XPS spectra of 2H-MoS_2 and *R/S/rac*-MBA_MoS_2

	Peak position (eV)	
	2H-MoS_2	*R/S/rac*-MBA_MoS_2
Mo^{4+} $3d_{5/2}$ (2H)	229.2	229.4
Mo^{4+} $3d_{3/2}$ (2H)	232.3	232.5
Mo^{4+} $3d_{5/2}$ (1T')	0	228.5
Mo^{4+} $3d_{3/2}$ (1T')	0	231.7
S $2p_{3/2}$ (2H)	162.0	162.5
S $2p_{1/2}$ (2H)	163.2	163.7
S $2p_{3/2}$ (1T')	0	161.3
S $2p_{1/2}$ (1T')	0	162.6

Table S2. The quantitative analysis of each peak area of 2H-MoS_2 and *R/S/rac*-MBA_MoS_2

		2H-MoS_2	*R*-MBA_MoS_2	*S*-MBA_MoS_2	*rac*-MBA_MoS_2
Quantitative Analysis/%	O	7.0	13.1	33.7	11.8
	N	0.0	0.3	0.6	0
	C	46.3	20.6	11.0	11.1
	Mo	15.4	20.3	14.6	24.0
	S	31.3	45.8	38.9	53.1
Mo 3d/%	Mo^{6+}	8	6	8	5
	2H-MoS_2	92	41	40	40
	1T'-MoS_2	0	53	53	55
S 2p/%	2H-MoS_2	100	32	35	31
	1T'-MoS_2	0	66	46	67

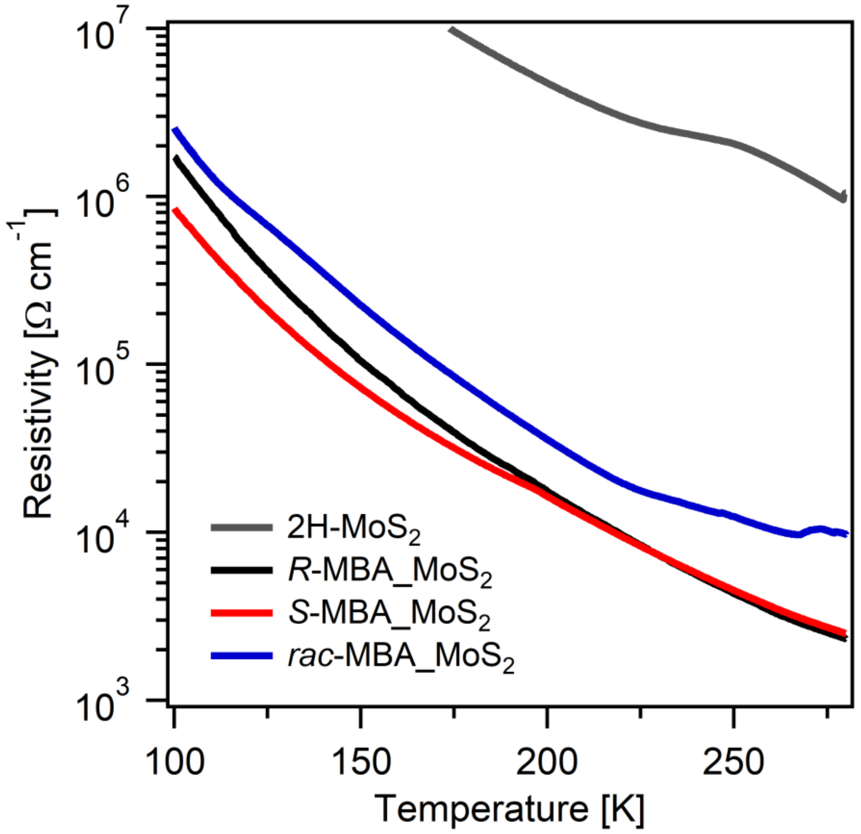

Figure S2. Temperature dependence of the electrical resistivity of *R/S/rac*-MBA_MoS_2 and 2H-MoS_2.

Table S3. The inter-layer distances between two molybdenum atoms of left and right MoS_2 monolayers and total energies of different strucutures, ΔE (The energy difference with the structure for horizontal-type or vertical-type which holds lowest total energy), as well as the preferred modes compared with horizontal-type and vertical-type for the same monolayer MoS_2 and MBA molecules.

Materials	Layer distance (Å)	Total energy (eV)	ΔE-horizontal (eV)	ΔE-vertical (eV)
2H-MoS_2	6.66	-60378.47		
2H-R-horizontal	9.49	-62147.03	0	
2H-R-vertical	14.50	-62145.72		0.05
2H-S-horizontal	9.95	-62146.74	0.29	
2H-S-vertical	14.38	-62145.74		0.03
2H-rac-horizontal	9.75	-62146.83	0.2	
2H-rac-vertical	14.05	-62145.77		0
1T'-MoS_2	6.14	-80487.80		
1T'-R-horizontal	9.55	-82253.34	0	
1T'-R-vertical	13.79	-82251.55		0.30
1T'-S-horizontal	9.66	-82253.03	0.31	
1T'-S-vertical	13.91	-82251.85		0
1T'-rac-horizontal	9.86	-82252.48	0.86	
1T'-rac-vertical	13.89	-82251.58		0.27

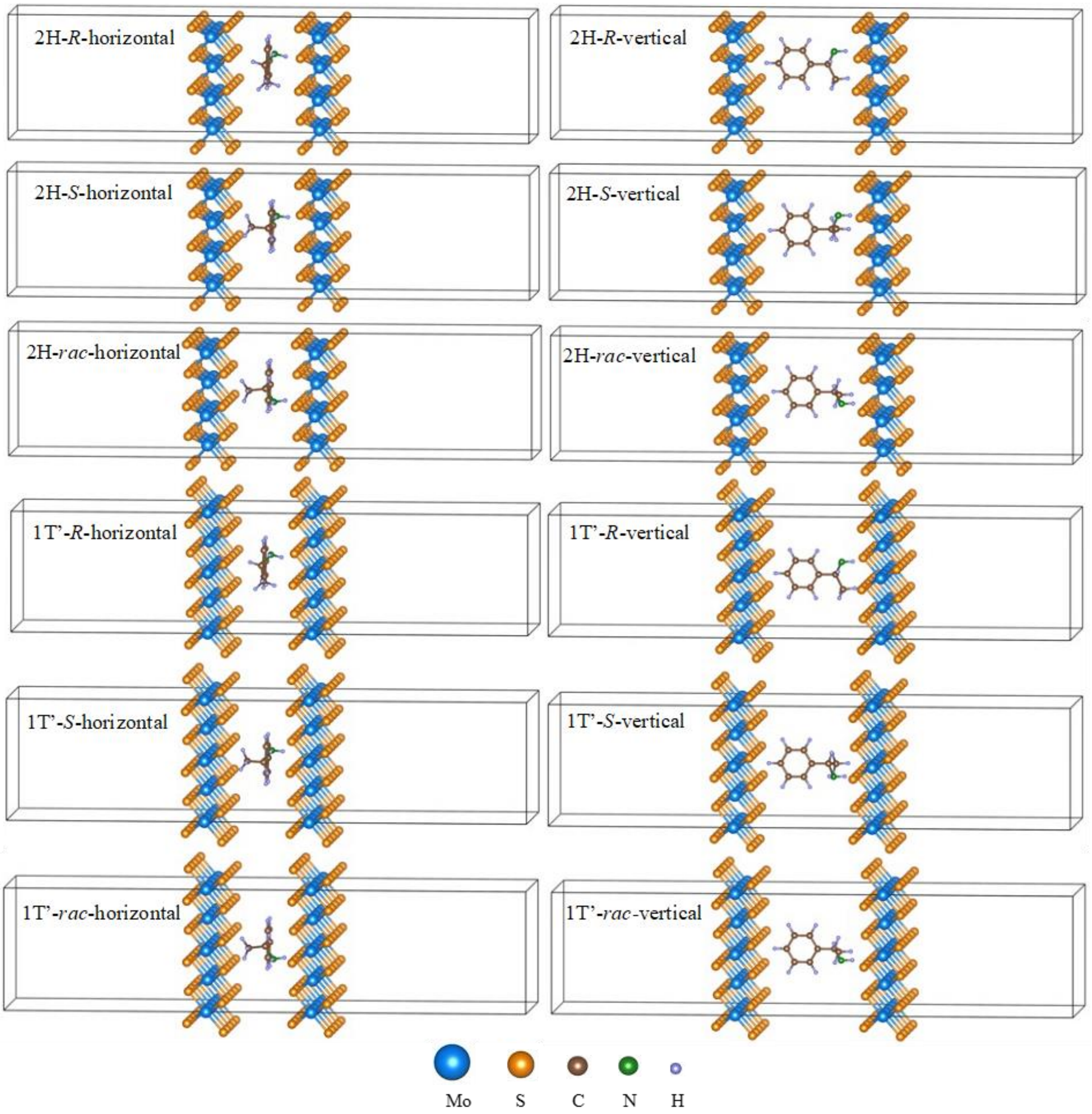

Figure S3. The schematic diagram of "MoS_2/MBA/MoS_2" structures, the blue and yellow balls represent Molybdenum and Sulfur atoms, the brown, green and purple balls represent nitrogen, hydrogen and carbon atoms. The layer distances are between two molybdenum atoms of left and right MoS_2 monolayers.

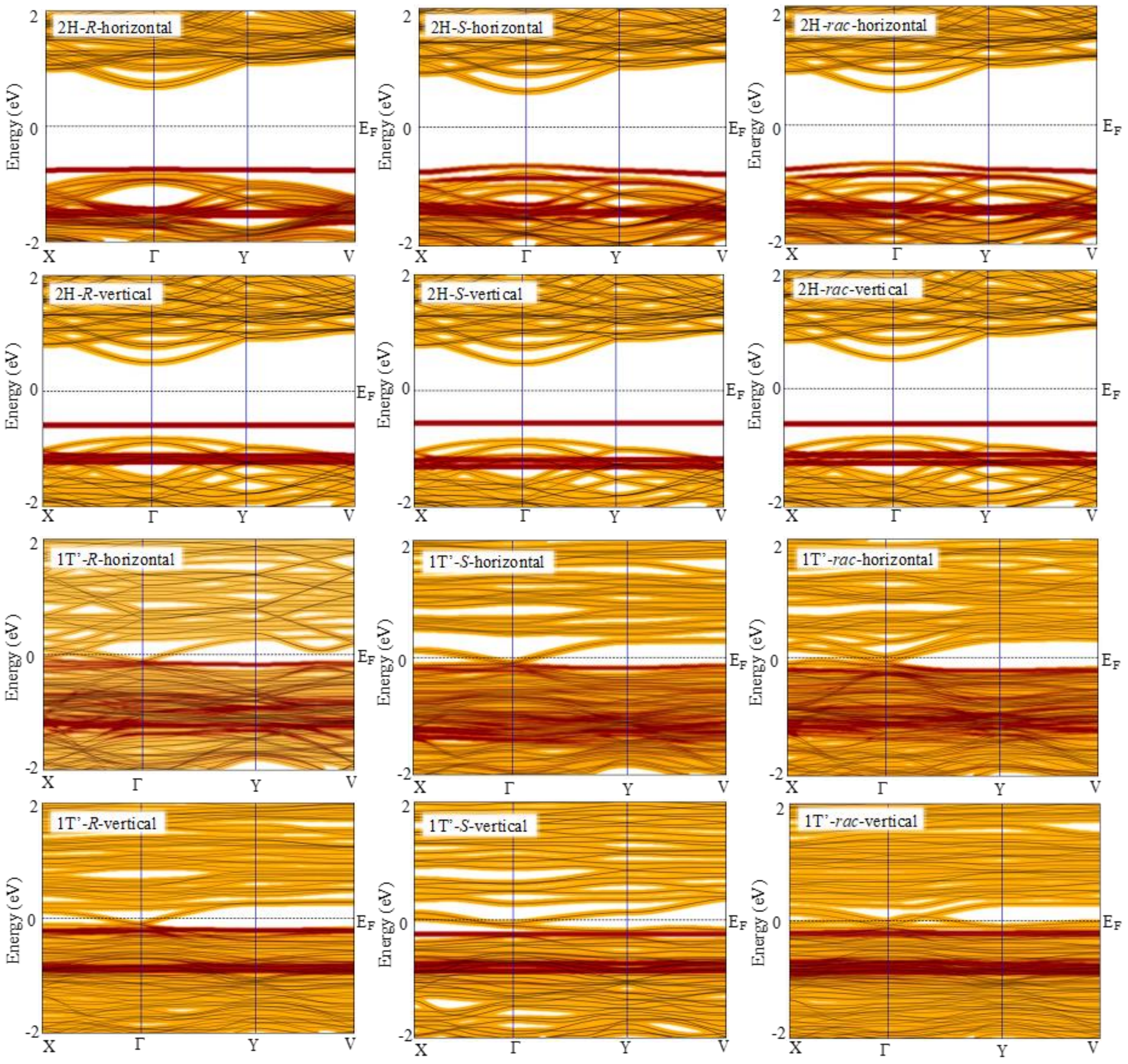

Figure S4. The fat bandstructures of "MoS_2/MBA/MoS_2" optimized structures. Red and organge lines represent the contribution from MBA molecules and MoS_2, respectively.

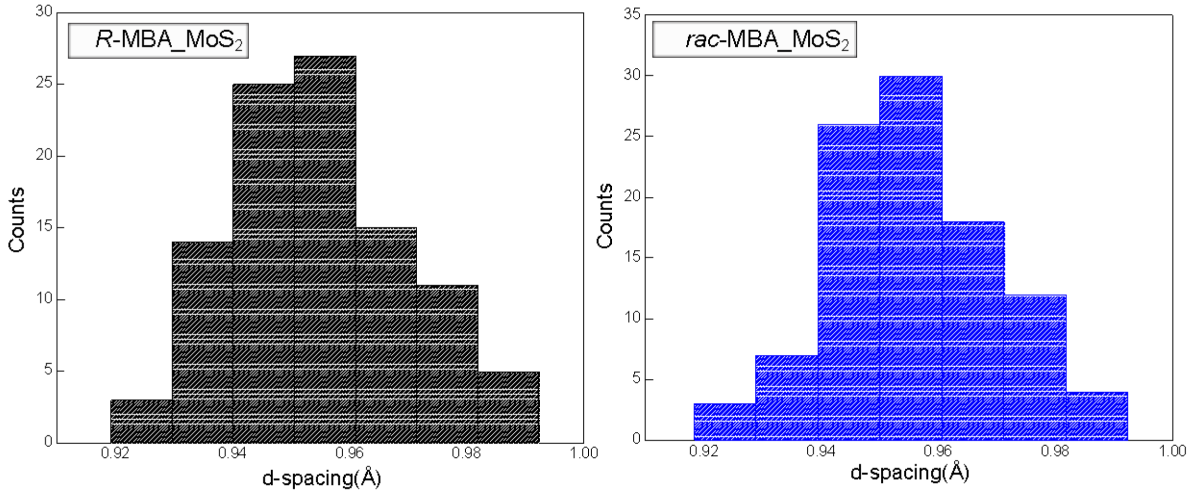

Figure S5. The histograms of inter-layered distance distribution of the R-MBA_MoS_2 and rac-MBA_MoS_2 from collected over 100 different positions.

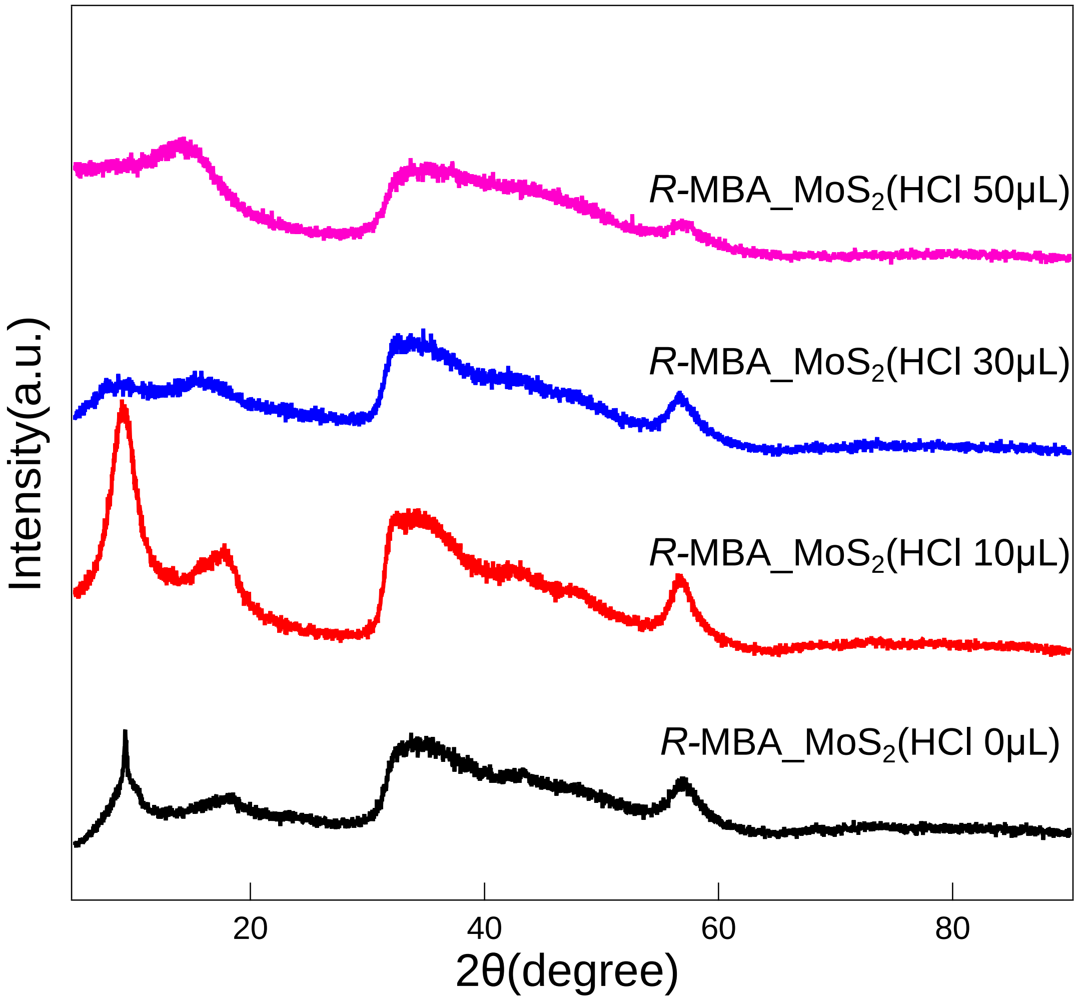

Figure S6. The XRD patterns of *R*-MBA_MoS_2 obtained in different HCl solutions.

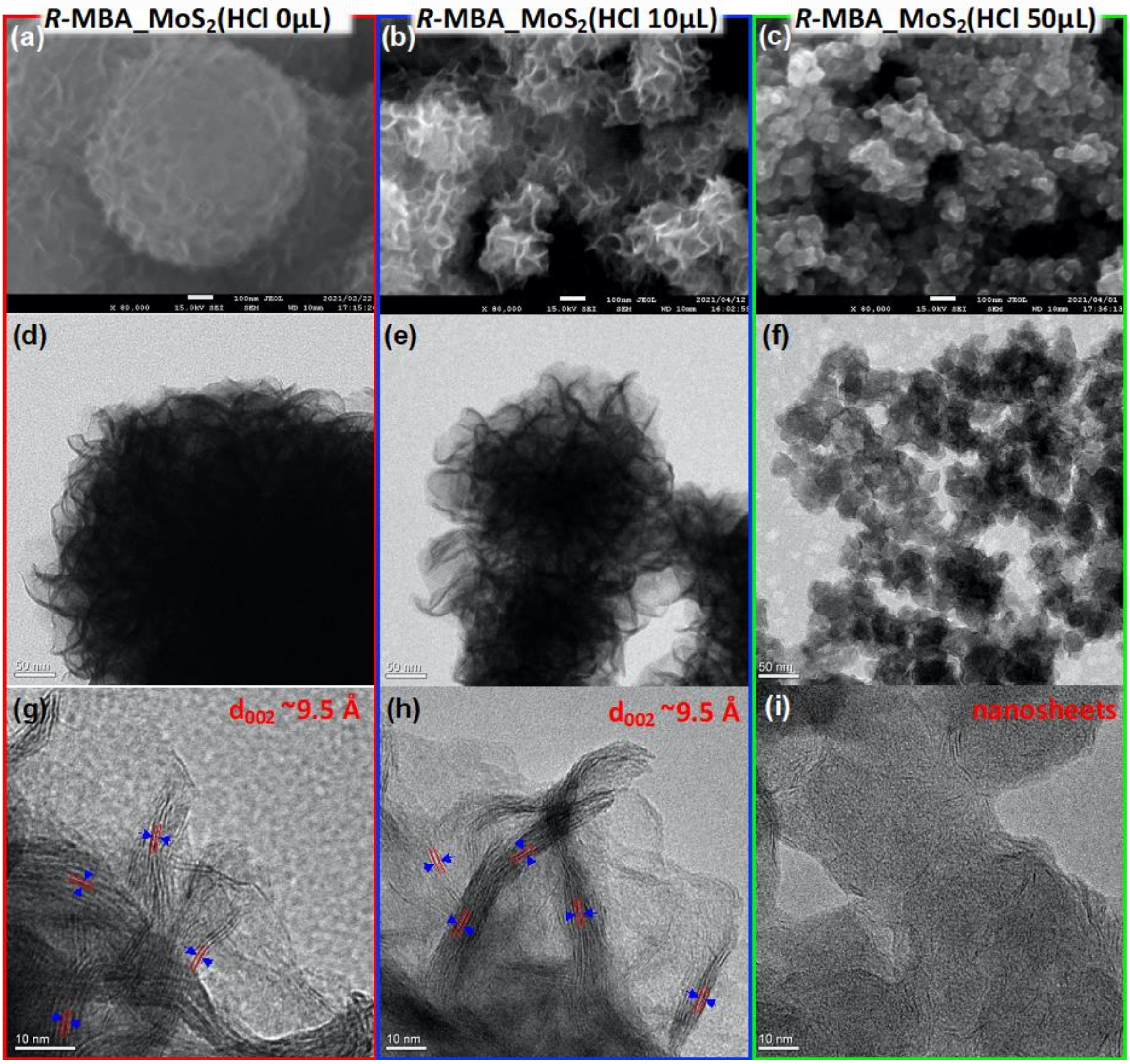

Figure S7. The (a-c) SEM, (d-f) TEM and (g-i) HRTEM images in basic (HCl 0μL), neutral (HCl 10μL) and acidic (HCl 50μL) solutions of *R*-MBA_MoS_2.

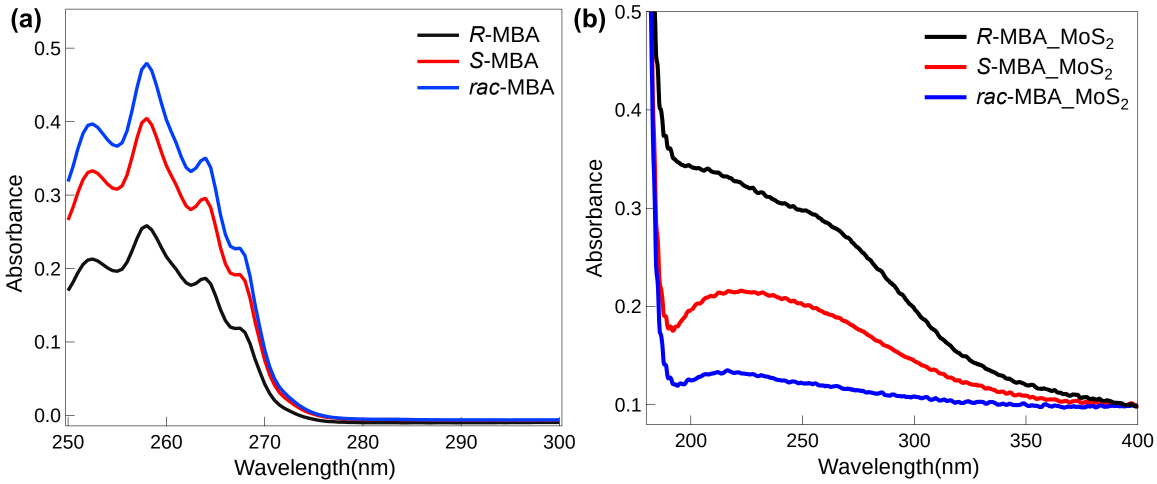

Figure S8. The electronic absorption spectra of (a) *R/S/rac*-MBA solutions and (b) *R/S/rac*-MBA_MoS_2 thin films, respectively.

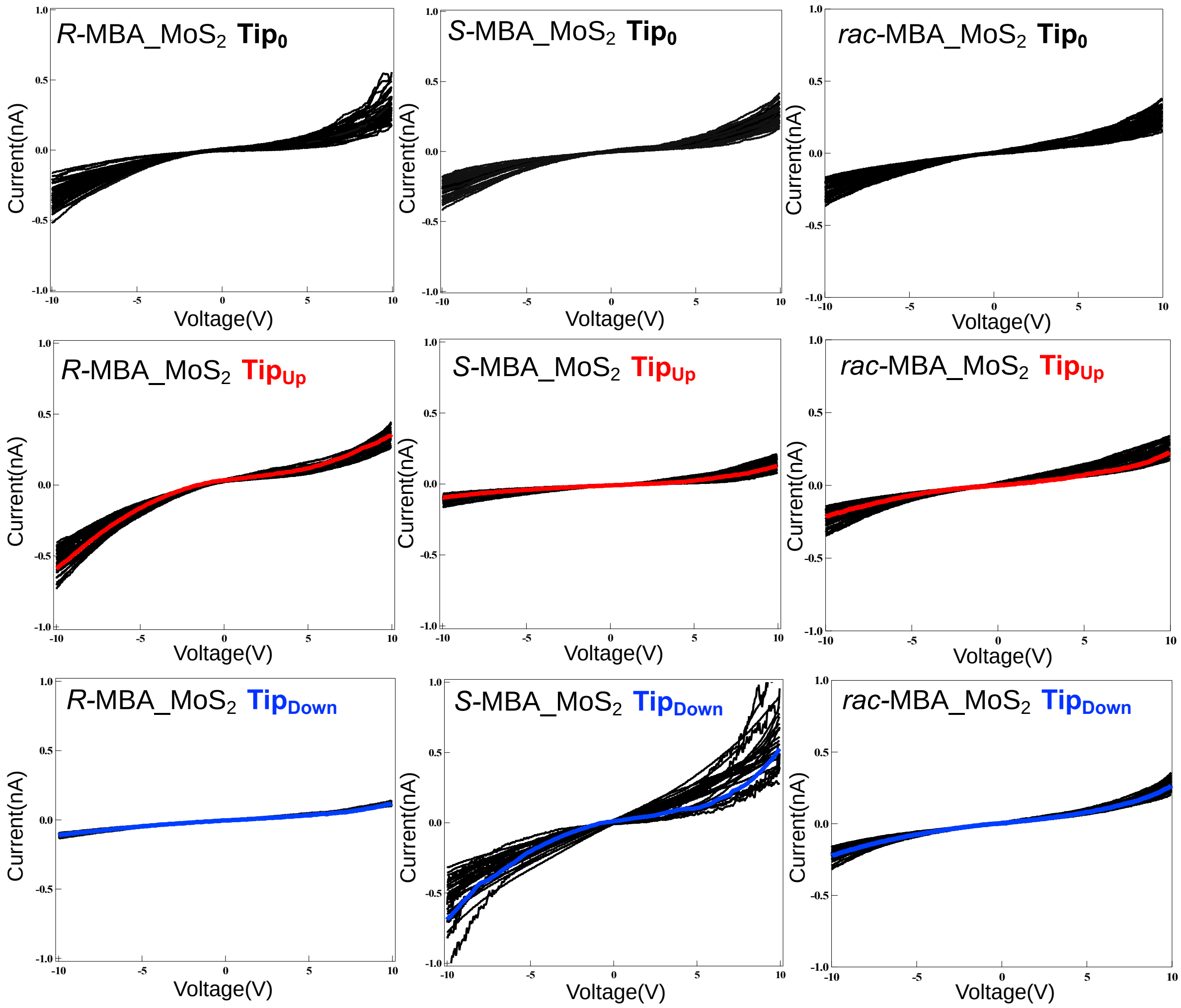

Figure S9. *I*-*V* curves (nonaveraged raw data) recorded by the spin-polarized conductive AFM measurements with tip magnetized along up or down magnetic field orientation at different position s of the tip on each sample.

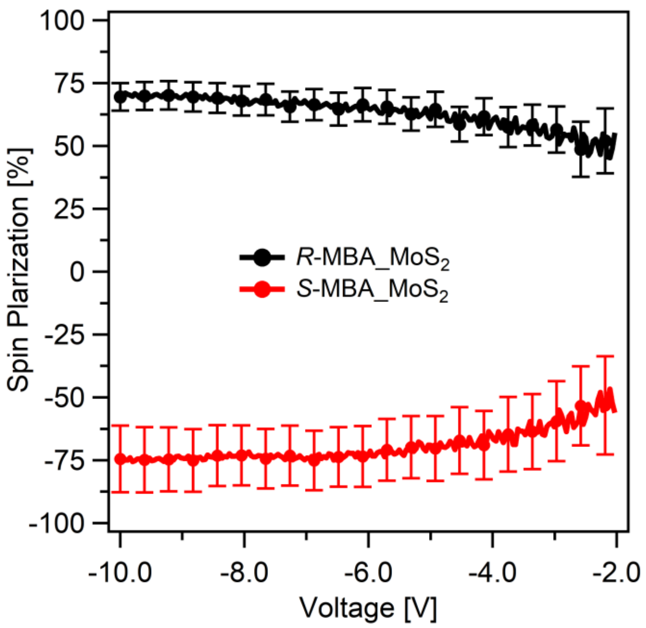

Figure S10. Spin polarization as a function of applied bias for *R*-MBA_MoS_2 and *S*-MBA_MoS_2 from -10 to -2 V. Error bars indicate standard deviations.

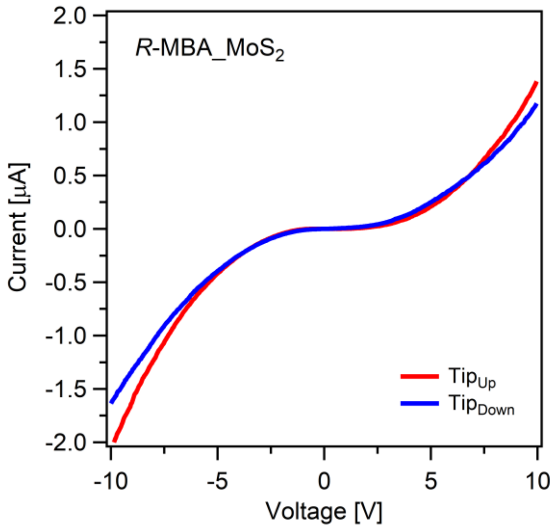

Figure S11. Spin-polarized c-AFM measurements. *I–V* curves from -10 to +10 V of the chiral *R*-MBA_MoS_2 thin films (ca. 1.5 μm). The CoCr tip used was magnetized along the upward (red), or downward (blue) orientations. The average *I–V* curve recorded over 40 scans at different points is shown. The *SP* is calculated for ~16%.

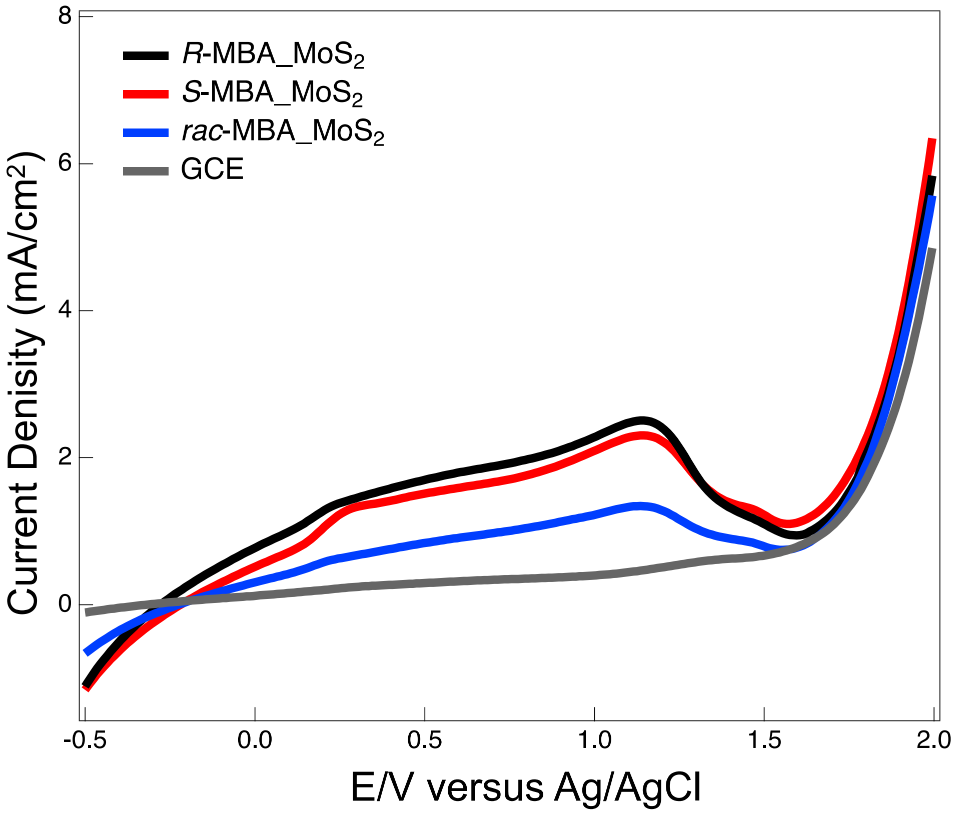

Figure S12. The linear sweep voltammetry (LSV) curves for *R/S/rac*-MBA_MoS_2 and bare glassy carbon electrode (GCE) electrodes in a 0.1 M Na_2SO_4 (pH 6.56) aqueous electrolyte solution, with a scan rate of 10 mV/s.

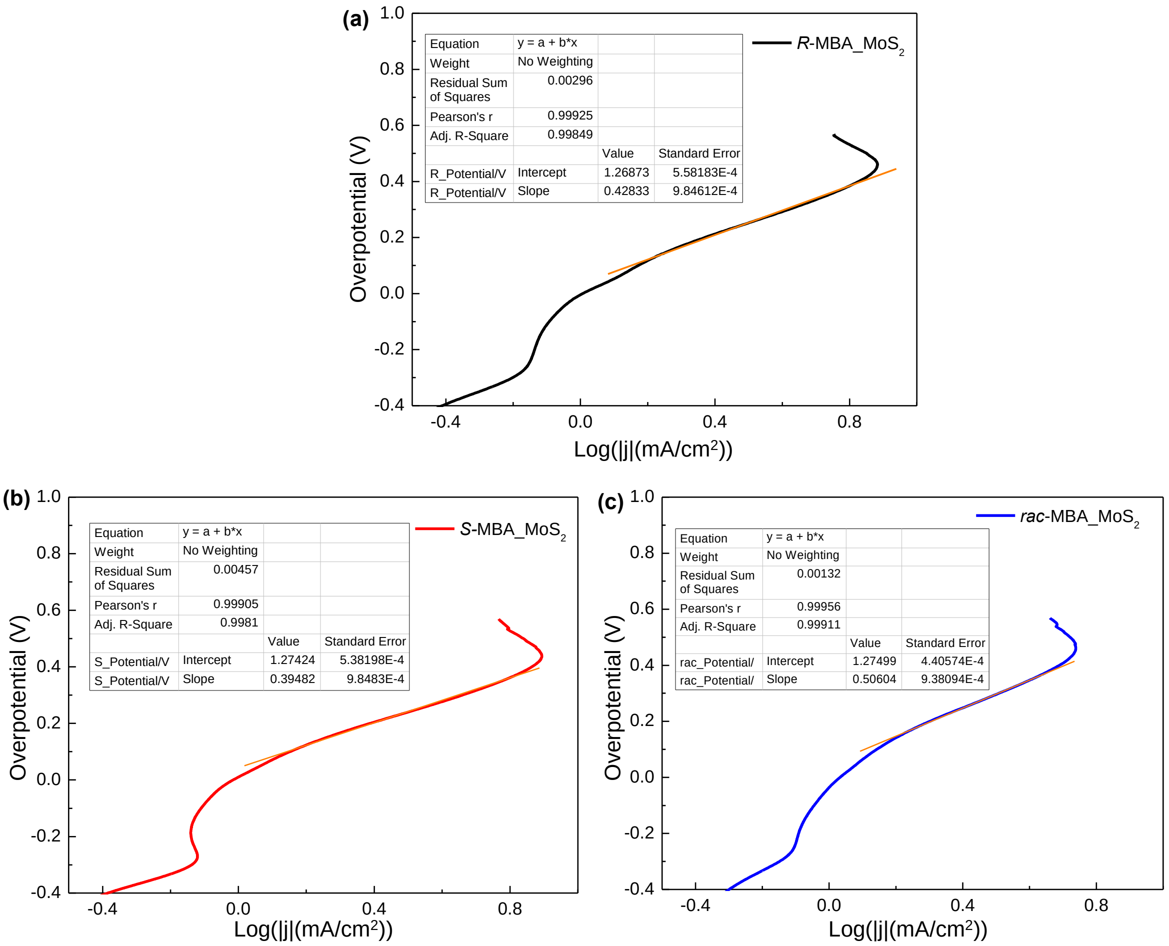

Figure S13. Tafel plots from the LSV data are shown for the (a) *R*-MBA_MoS_2 (b) *S*-MBA_MoS_2 (c) *rac*-MBA_MoS_2 electrodes. The orange lines are the linear fitting curves.

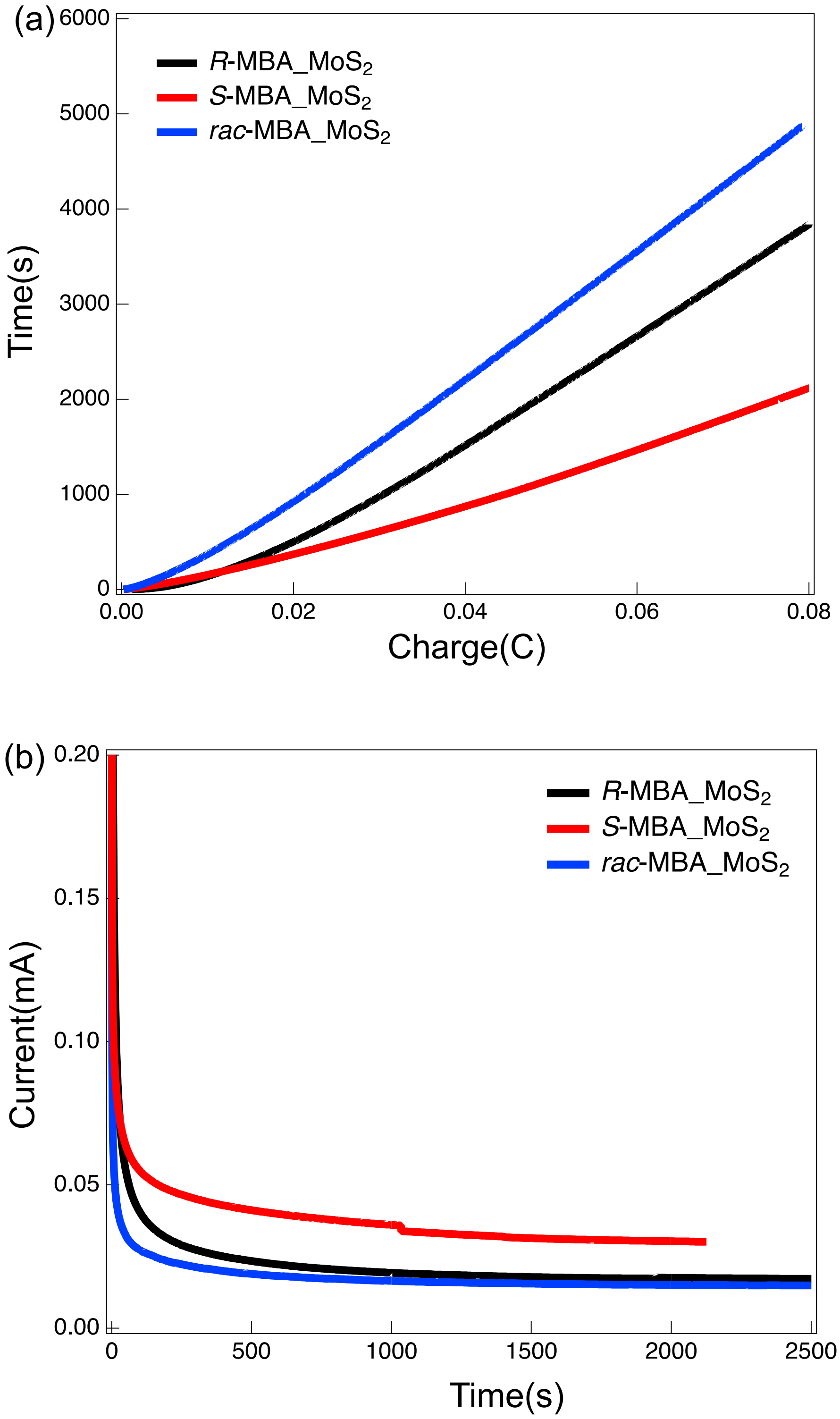

Figure S14. (a) Time as a function of charge and (b) current as function of time on *R*-MBA_MoS_2, *S*-MBA_MoS_2 and *rac*-MBA_MoS_2 electrodes to quantify the amount of H_2O_2 produced.

(a) (b)

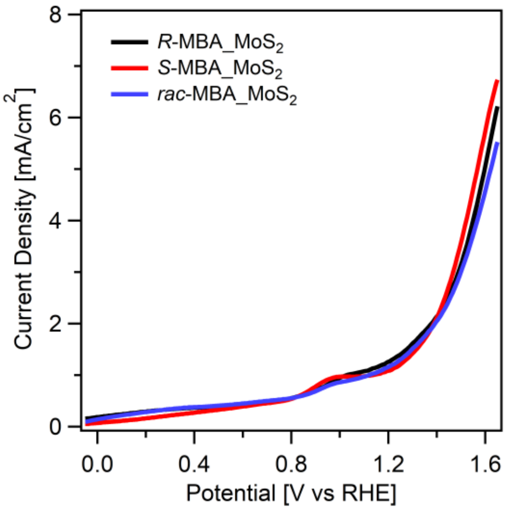

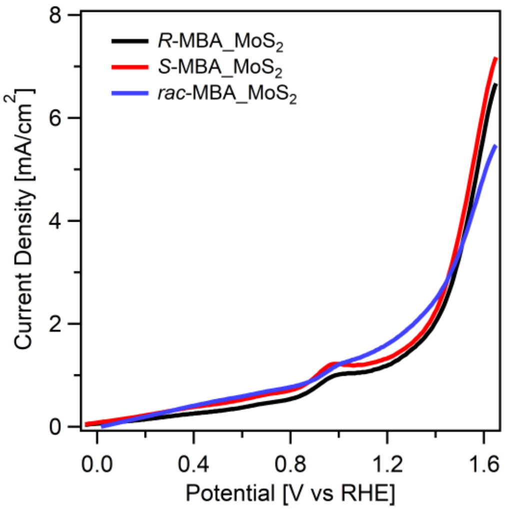

Figure 15. Performance of the *R/S/rac*-MBA_MoS$_2$ samples as electrocatalysts for OER. LSV curves from 0 to 1.6 V in 0.1 M KOH recorded at a scan rate of 10 mV s^{-1} for the sample 2 (a) and the sample 3 (b).

(a) **(b)**

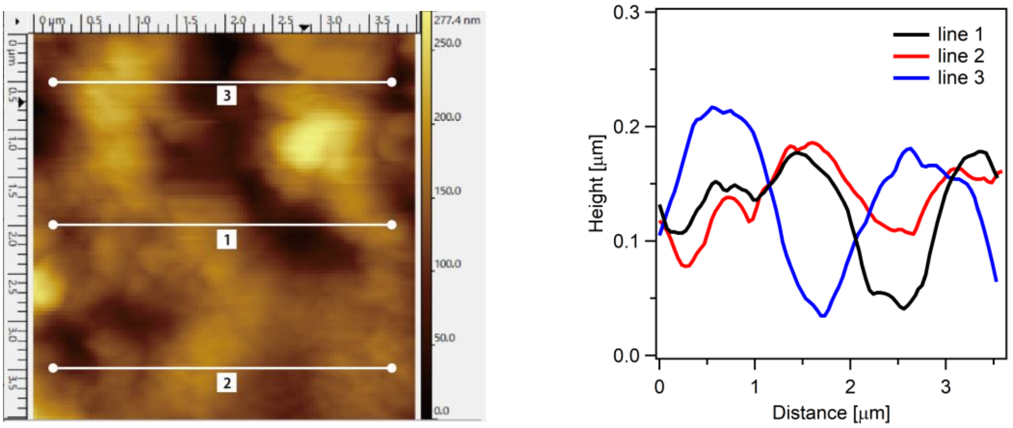

Figure S16. (a) typical AFM image for the typical MBA_MoS_2 films used for spin-polarized c-AFM measurements and (b) the corresponding cross-sectional profiles.
